# The GABA transaminase GabT is required for full virulence of *Ralstonia solanacearum* in tomato

**DOI:** 10.17912/micropub.biology.000478

**Published:** 2021-09-29

**Authors:** Liu Xian, Gang Yu, Alberto P Macho

**Affiliations:** 1 Shanghai Center for Plant Stress Biology, Center for Excellence in Molecular Plant Sciences, Chinese Academy of Sciences

## Abstract

The utilization of plant gamma-aminobutyric acid (GABA) is essential for the pathogenicity of the bacterial plant pathogen *Ralstonia solanacearum*. A knockout mutant in the GABA transaminase-encoding gene *gabT* is unable to utilize GABA as a nutrient and its ability to cause disease in plants is strongly compromised. However, the proximity of the *gabD* gene (encoding a succinate semialdehyde dehydrogenase) in the same operon raises the question of whether an impact on the *gabD* gene underlies or contributes to the virulence attenuation of the *ΔgabT* mutant. In this work, we use genetic complementation to show that the expression of the *gabT *gene is able to rescue the impaired virulence of the *ΔgabT* knockout mutant in tomato plants, confirming that the *gabT*-encoded GABA transaminase is indeed required for full virulence of *R. solanacearum* in a natural host plant.

**Figure 1. Expression of gabT complements the virulence attenuation of the ΔgabT mutant:**
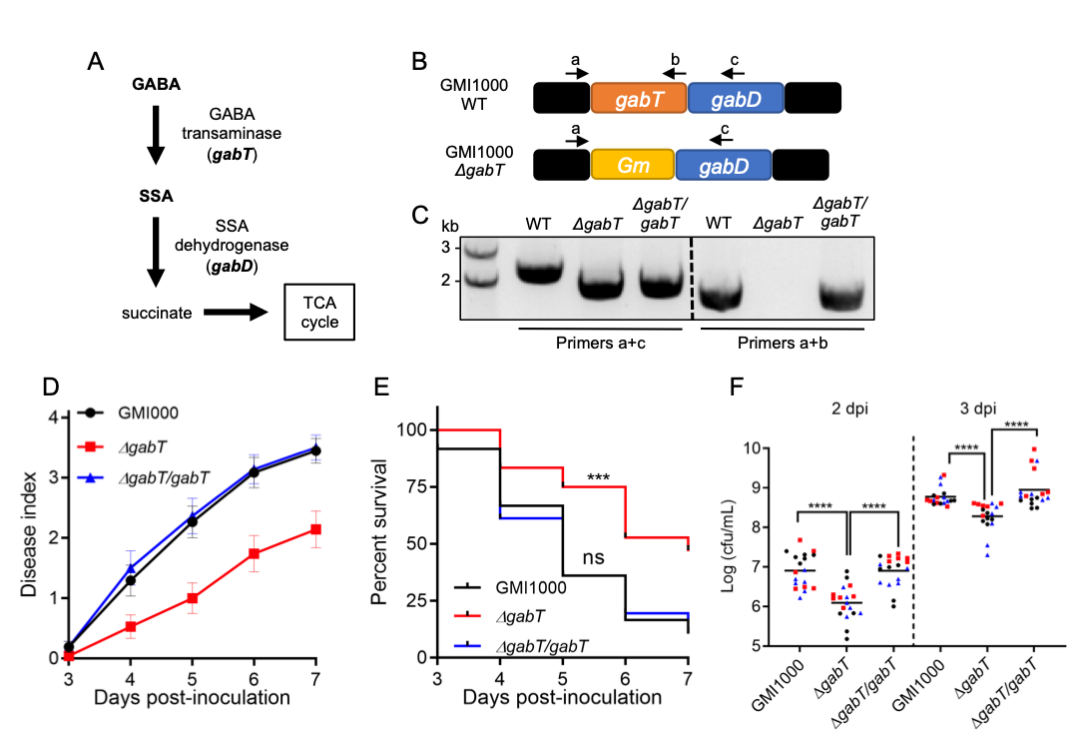
**A.** Predicted GABA catabolic pathway in *R. solanacearum*. GABA is converted into succinate semialdehyde (SSA) by GABA transaminases (GabT); SSA is used by succinate semialdehyde dehydrogenases (GabD) to generate succinate, which is eventually incorporated into the TCA cycle. **B.** Structure of the *gabT-gabD* operon in *R. solanacearum* GMI1000 (the *gabT* gene is also called *goaG*; Salanoubat *et al.*, 2002). In the *ΔgabT* mutant, a Gentamycin (Gm) resistance cassette replaces the genomic sequence of the *gabT* gene. Arrows show the hybridization sites of the primers used in (C) for the validation of these strains. **C.** Validation of the *ΔgabT* mutant and *ΔgabT*/*gabT* complementation strains by PCR. Primers hybridizing in the *gabT* promoter and the *gabT/gabD* genes were used. The predicted size of the resulting fragment using primers a+c in a strain containing the original *gabT* allele (only present in the WT strain) is 2166 bp, while the predicted size of the fragment containing the Gm resistance cassette is 1760 bp. The predicted size of the resulting fragment using primers a+b (amplifying the *gabT* gene in both WT strain and complementation strain) is 1581 bp. Marker sizes are shown for reference. **(D and E)** Soil drenching inoculation assays in tomato Moneymaker plants using *R. solanacearum* GMI1000, a *gabT* mutant (*ΔgabT*), and a GabT complementation strain (*ΔgabT/gabT*). In **(D)** the results are represented as disease progression, showing the average wilting symptoms in a scale from 0 to 4. Values from 3 independent biological repeats were pooled together (mean ± SEM; n=36). **(E)** Survival analysis of the data in (D); the disease scoring was transformed into binary data with the following criteria: a disease index lower than 2 was defined as ‘0’, while a disease index equal or higher than 2 was defined as ‘1’ for each specific time point. Statistical analysis was performed using a Log-rank (Mantel-Cox) test. **(F)** Growth of *R. solanacearum* GMI1000 WT*, ΔgabT*, and *ΔgabT/gabT* in tomato stems. Five μL of bacterial suspension (10^6 ^cfu mL^-1^) were injected into the stem of 4-week-old tomato plants and xylem sap was extracted from each infected plant for bacterial quantification 2 or 3 days post-inoculation (dpi). Different colours represent values obtained in 3 independent biological repeats, and horizontal bars represent average values (n=6 plants per strain in each repeat). Statistical analysis was performed using a Student’s *t* test. In E and F, asterisks indicate significant differences using the indicated statistical tests (ns, not significant, *** p<0.001, **** p<0.0001).

## Description

*Ralstonia solanacearum* is the causal agent of bacterial wilt disease of more than 250 plant species, and is considered as one of the most destructive plant pathogens (Mansfield *et al.*, 2012). *R. solanacearum* is a soil-borne bacterium that enters plants through the roots, eventually reaching the vascular system; bacterial proliferation in xylem vessels causes vascular blockage, which leads to plant wilting and death (Xue *et al.*, 2020). We have recently shown that *R. solanacearum* promotes the production of gamma-aminobutyric acid (GABA) in plant cells, which in turn can be used as a nutrient to support bacterial replication and pathogenesis inside the plant (Xian *et al.*, 2020). In other gram-negative bacteria, such as *R. eutropha* (also known as *Cupriavidus necator*)or *Pseudomonas syringae*, GABA catabolism involves GABA transaminases (GabT), which convert GABA into succinate semialdehyde (SSA); SSA is used by succinate semialdehyde dehydrogenases (SSA-DH / GabD) to generate succinate, which can be incorporated into the TCA cycle (Lutke-Eversloh & Steinbuchel, 1999; Park *et al.*, 2010; Figure 1A). The genome of *R. solanacearum* contains a single copy of the *gabT* gene (RSc0029), which is located upstream of the *gabD* gene (RSc0028) in the same operon (Figure 1B). Interestingly, a *ΔgabT* knockout mutant was unable to use GABA as a nutrient, and severely impaired in its ability to cause disease symptoms in the model plant *Arabidopsis thaliana* and to grow in stems of tomato plants, their natural host (Xian *et al.*, 2020). These results suggest that *R. solanacearum* requires the GabT transaminase to cause disease. However, given that the *gabT* and *gabD* genes belong to the same operon, it is formally possible that the mutation in *gabT* affects the expression of *gabD*, which could also contribute to the reduced virulence observed in the *ΔgabT* mutant. In order to address this possibility, we complemented the *ΔgabT* mutant using an integrative plasmid that leads to the expression of the *gabT* gene driven by its own promoter (see methods; Figure 1C). First, we inoculated the *ΔgabT* mutant in tomato plants by soil-drenching, an inoculation method that mimics the natural infection route of *R. solanacearum*, and found that, as previously shown in Arabidopsis, the *ΔgabT* mutant is significantly impaired in its ability to cause disease symptoms in tomato, in comparison to the GMI1000 wild-type reference strain (Figure 1D and 1E). Importantly, the symptoms induced by the complementation strain expressing *gabT* in the *ΔgabT* mutant background (*ΔgabT*/*gabT*) were similar to those induced by the GMI1000 wild-type strain (Figure 1D and 1E). Moreover, upon injection in tomato stems, expression of the *gabT* gene was able to rescue the attenuation in growth observed in the *ΔgabT* mutant (Figure 1F). Altogether, these results indicate that the virulence attenuation observed in the *ΔgabT* mutant (Xian *et al.*, 2020; Figure 1D and 1E) is indeed caused by mutation of the *gabT* gene. These results also suggest that either the *gabD* gene is still expressed in the *ΔgabT* mutant, or the *gabD* gene is not required for virulence. However, if the *gabD* gene is not required for virulence, how does *R. solanacearum* catabolize SSA to complete the GABA catabolic pathway? In *R. eutropha* it has been shown that a *gabD* mutant still displays SSA-DH activity (Lutke-Eversloh & Steinbuchel, 1999), suggesting the presence of additional SSA-DH enzymes, and such non-GabD SSA-DH enzymes have been identified in other bacteria, such as *Escherichia coli* or *Klebsiella pneumoniae* (Marek & Henson, 1988; Sanchez *et al.*, 1989). Similarly, even if the *gabD* gene were affected in the *ΔgabT* mutant, it is possible that *R. solanacearum* uses other SSA-DH enzymes to complete the GABA catabolic pathway in the *ΔgabT*/*gabT* complementation strain.

## Methods

**Plant material and growth conditions**

***Solanum lycopersicum***

Tomato plants (*Solanum lycopersicum* cv. Moneymaker) were cultivated in jiffy pots (Jiffy International, Kristiansand, Norway) in a growth chamber under controlled conditions (25°C, 16 h light/8 h dark photoperiod, 130 mE m^-2 ^s^-1^, 65% humidity) for 4 weeks. After soil drenching inoculation, the plants were kept in a growth chamber under the following conditions: 75% humidity, 12 h light, 130 mE m^-2 ^s^-1^, 27°C, and 12 h darkness, 26°C for disease symptom scoring.

**Bacterial strains**

*Ralstonia solanacearum* strains, including the phylotype I reference strain GMI1000, GMI1000 *ΔgabT* mutant, and GMI1000 *ΔgabT/gabT* complementation strains were cultured overnight at 28 °C in complete BG liquid medium (Plener *et al.*, 2010).

**Generation of *R. solanacearumgabT* complementation strain**

To complement *gabT* in the *ΔgabT* mutant (Xian *et al.*, 2020), the *gabT* gene (RSc0029) with its promoter (300 bp upstream of ATG of the *gabT* gene) was amplified by PCR, cloned into pENTR-TOPO, and then introduced into pRCT-GWY (Monteiro *et al.*, 2012) by LR reaction (in vitro recombination between the entry clone – pENTR-TOPO- and the destination vector – pRCT-GWY), resulting in the pRCT-*pgabT*–*gabT* (Vailleau *et al.*, 2007) plasmid. The integrative pRCT-*pgabT*–*gabT* plasmid, triggering the expression of *gabT* under the control of the native *gabT* promoter, was transformed into the *R. solanacearum* *ΔgabT* mutant strain (in GMI1000 background) by natural transformation, leading to the stable integration of the *pgabT-gabT* fragment into a permissive site of the bacterial chromosome, as previously described (Monteiro *et al.*, 2012). The complementation strain *ΔgabT*/*gabT* was selected using Tetracycline (10 μg mL^-1^) and confirmed by PCR using *gabT* specific primers (Figure 1b and Table S1).

**Pathogen inoculation assays**

For *R. solanacearum* soil-drenching inoculation, 12 3-4 week-old tomato plants for each bacterial strain (grown in Jiffy pots) were inoculated by soil drenching with a bacterial suspension containing 10^8^ colony-forming units per mL (CFU mL^-1^). 300 mL of inoculum of each strain was used to soak each set of plants. After 20-minute incubation with the bacterial inoculum, plants were transferred from the bacterial solution to a bed of potting mixture soil in a new tray (Vailleau *et al.*, 2007). Scoring of visual disease symptoms on the basis of a scale ranging from ‘0’ (no symptoms) to ‘4’ (complete wilting) was performed as previously described (Vailleau *et al.*, 2007).

Stem injection assays with *R. solanacearum* were performed as previously described (Yu *et al.*, 2020; Wang *et al.*, 2021a; Wang *et al.*, 2021b). Briefly, 5 μL of a 10^6^ CFU mL^-1^ bacterial suspension were injected into the stems of 4-week-old tomato plants and 2.5 μL xylem sap were collected from each plant for bacterial quantification 2 or 3 days-post inoculation (dpi). Injections were performed 2 cm below the cotyledon emerging site in the stem, while the samples were taken at the cotyledon emerging site. CFUs were counted by spreading serial dilutions on solid BG medium (Plener *et al.*, 2010).
